# Neurotoxic Metal Coexposures: Claus Henn et al. Respond

**DOI:** 10.1289/ehp.1205004R

**Published:** 2012-06-01

**Authors:** Birgit Claus Henn, David C. Bellinger, Robert O. Wright

**Affiliations:** Department of Environmental Health, Harvard School of Public Health, Boston, Massachusetts, E-mail: bclaus@hsph.harvard.edu

We thank Dórea for his comments on the importance of examining breast-feeding and ethylmercury exposure in our study of manganese–lead coexposures and neurodevelopment (Claus Henn et al. 2012). We agree that both breast-feeding and organic mercury exposure may affect neurodevelopment and have the potential to act as confounders and/or effect modifiers in analyses  of metal effects on neurodevelopment.

To be a confounder, a variable must be associated with both exposure and outcome. In our data, duration of breast-feeding was not strongly associated with exposures (measured by blood manganese and lead levels). When breast-feeding variables were forced into final models, the manganese–lead effect estimates did not change appreciably. Although breast-feeding did not appear to be an important confounder in our data, we agree with Dórea that this factor needs to be considered in studies of prenatal and early life environmental exposures and neurodevelopment.

We agree with Dórea that organic mercury  may be an important coexposure, acting potentially as a confounder and/or effect modifier of the manganese–lead association with neurodevelopment. We do not have detailed data on vaccination rates and ages. However, if the primary source of mercury exposure among these participants is via thimerosal-containing vaccines, as Dórea suggests, then in order for mercury to be a confounder, becoming vaccinated must be associated with manganese and lead exposures. We posit that any vaccination and lead–manganese exposure association would be weak at best, thereby reducing concerns that confounding by ethylmercury would explain observed associations between manganese–lead exposure and neurodevelopment. Nonetheless, we recognize that exposure to mercury and other co-pollutants may be a source of unmeasured confounding, which we stated in our article (Claus Henn et al. 2012).

Three-way interactions are undoubtedly important and warrant attention. The limited sample size in our study population precludes us from looking at a manganese–lead–mercury interaction. In the future, epidemiologic studies should be designed with sufficient power to address the impact of multiple metal exposures, including three-way interactions.
